# Diagnostic Significance of Selected Serum Inflammatory Markers in Women with Advanced Endometriosis

**DOI:** 10.3390/ijms22052295

**Published:** 2021-02-25

**Authors:** Izabela Kokot, Agnieszka Piwowar, Marcin Jędryka, Katarzyna Sołkiewicz, Ewa Maria Kratz

**Affiliations:** 1 Department of Laboratory Diagnostics, Division of Laboratory Diagnostics, Faculty of Pharmacy, Wroclaw Medical University, Borowska Street 211A, 50-556 Wroclaw, Poland; katarzyna.solkiewicz@umed.wroc.pl (K.S.); ewa.kratz@umed.wroc.pl (E.M.K.); 2 Department of Toxicology, Faculty of Pharmacy, Wroclaw Medical University, Borowska Street 211A, 50-556 Wroclaw, Poland; agnieszka.piwowar@umed.wroc.pl; 3 Department of Oncology, Gynecological Oncology Clinic, Faculty of Medicine, Wroclaw Medical University, Hirszfeld Square 12, 53-413 Wroclaw, Poland; marcin.jedryka@umed.wroc.pl; 4 Department of Oncological Gynecology, Wroclaw Comprehensive Cancer Center, Hirszfeld Square 12, 53-413 Wroclaw, Poland

**Keywords:** advanced endometriosis, serum parameters of inflammation, cytokines, CA 125, diagnostics of endometriosis

## Abstract

Endometriosis is a gynecological disease, the pathogenesis of which seems to be directly associated with inflammatory processes. Serum concentrations of IL-1β, IL-6, hs-CRP, IgG, YKL 40 and PRL, in comparison to the well-known CA 125 levels, were studied with the aim of identifying an additional noninvasive inflammatory marker or set of markers characteristic for endometriosis. The study group included 43 women with endometriosis (E), 35 women with benign gynecological disorders but without endometriosis (NE, non-endometriosis) as a comparative group, and a control group consisting of 18 healthy subjects (C). The serum concentrations of IL-1β, IL-6, hs-CRP, YKL-40, PRL and CA 125 were significantly higher in the E group (median values: 0.41 pg/mL, 2.42 pg/mL, 2.33 mg/L, 79.30 ng/mL, 21.88 ng/mL and 68.00 U/mL, respectively) than in the control group (median values: 0.21 pg/mL, 0.98 pg/mL, 0.52 mg/L, 49.77 ng/mL, 12.08 ng/mL and 12.20 U/mL respectively), with the significance of *p* = 0.011, *p* < 0.001, *p* = 0.028, *p* = 0.005, *p* < 0.001 and *p* < 0.001, respectively. The IgG concentrations were significantly lower in the endometriosis group (median value: 1061.21 mg/dL) as compared to healthy women (median value: 1210.50 mg/dL; *p* = 0.025). Significant differences in concentrations of IL-6 (*p* = 0.040), hs-CRP (*p* = 0.007) and CA 125 (*p* < 0.001) were observed in stage III vs. stage IV of endometriosis. Significantly higher concentrations of IL-6 (*p* = 0.010), hs-CRP (*p* = 0.037) and PRL (*p* < 0.001) were observed in the NE group vs. the control group. Only CA 125 concentrations were significantly higher in endometriosis patients as compared to the non-endometriosis group (*p* < 0.001). The proposed panel of inflammatory markers, especially IL-6, PRL and CA 125, may become a useful tool to identify women with advanced endometriosis who could qualify for treatment.

## 1. Introduction

Endometriosis is quite a common gynecological disease, which is difficult to diagnose and treat. It affects about 10% of young women, which represents approximately 200 million women of reproductive age [1]. That number increases up to 50% in the group of patients with chronic pelvic pain, infertility, or both [2]. The common symptoms of endometriosis are dysmenorrhea, dyspareunia and back pain, as well as bladder and/or bowel problems, which may vary in degree and intensity [1]. These symptoms are usually nonspecific, which makes the clinical diagnosis of the disease very difficult [3]. The gold standard for the diagnosis of endometriosis remains laparoscopy, which delivers both clinical and histological findings. However, the procedure is invasive and many patients are not willing to have it performed as soon as the disease is suspected. This, in turn, can delay diagnosis by as long as 9 years [4]. In some types of endometriosis, imaging techniques (e.g., ultrasonography, magnetic resonance imaging) are useful and strongly recommended for correct diagnosis [3]. On the other hand, superficial peritoneal endometriosis is hard to detect with the use of non-invasive procedures [4]. Moreover, even such renowned standards for endometriosis diagnostics as laparoscopy may fail in the very early stages of the disease [5].

Endometrial tissues are presented and developed in the ectopic site, especially in the pelvis. Endometrial infiltrates are most often found in the ovaries, peritoneum, uterosacral ligaments, pouch of Douglas and rectovaginal septum [6]. Sometimes, however, the changes occur far beyond the pelvic organs, e.g., in the lungs, pleura, spinal cord, nose and abdominal lymph nodes [2]. Based on the severity and anatomical extension of the lesions, endometriosis is graded into four stages: minimal, mild, moderate and severe—in accordance with the revised American Fertility Society (rAFS) classification [7], which was updated by the American Society for Reproductive Medicine (ASRM) [8].

It has previously been suggested that the etiology of endometriosis is related to retrograde menstruation, during which endometrial cells are placed in ectopic sites [6,9]. Sampson [10] was the first to describe this process, and his theory of endometriosis pathogenesis is the most probable [11]. However, not every woman with retrograde menstruation develops endometriosis. It seems obvious that other factors must also participate in the development and progression of the disease [12]. An induced immune system and inflammation may be among such factors, manifested by activated macrophages, T and B lymphocytes and cytokines [6]. It is suggested that cytokines in particular play a crucial role in the regulation of the whole process of the ectopic implantation of endometrial cells [2].

Previous studies by Patel et al. [2] suggested that patients with moderate to severe endometriosis have systemic but subtle immune dysregulation. These patients have impaired immune supervision and recognition of ectopic endometrial cells, secondary to defective NK-cell activity [2]. Moreover, IL-6 secreted by endometriotic cells, in connection with interferon-γ, may upregulate the production of sICAM-1 (the soluble form of intercellular adhesion molecule-1) by macrophages in patients with endometriosis. At the same time, increased secretion of sICAM-1 may allow endometrial fragments to evade immune surveillance, to survive and implant [6]. Many authors reported high levels of pro-inflammatory cytokines (e.g., IL-1β and IL-6 in pelvic fluid in women with endometriosis compared to the control group [2,13]), which indicates a significant role for immune markers in the pathogenesis of endometriosis. Endometriosis is also considered a local disease with systemic subclinical manifestation [14].

A widespread immunochemical marker of inflammatory conditions is C-reactive protein (CRP), which serves as a first-line marker of infection, inflammation or tissue damage in routine laboratory medicine. Since endometriosis is considered an inflammatory disease, CRP could be an additional potential non-invasive biomarker of it, however, a non-specific one. Very often, CRP concentration is measured using a highly sensitive method (hs-CRP), which detects low levels of it. Furthermore, CRP production is stimulated via pro-inflammatory cytokines, such as, e.g., IL-1 and IL-6 [14]. The body’s humoral immune response is based on the action of immunoglobulins (Ig) produced by plasma cells from the B-lymphocyte line [15]. Immunoglobulin G is the most frequent antibody class in the human serum and has the longest serum half-life. It is composed of four subclasses (IgG1–IgG4), with diverse functions. Nevertheless, the main roles of IgG are immune defense against various microorganisms and protection against the destruction of healthy tissues in autoimmune diseases [15,16]. Serum IgG concentration appears to be parallel to serum IL-6 concentration, as it shows the same upward trend [17].

One of the parameters of a chronic inflammatory condition is chitinase-3-like protein 1 (YKL-40) [18]. YKL-40 is a secretory protein that can act both locally, participating in intercellular signaling pathways, and systemically. This glycoprotein is produced by various cell types, e.g.,: macrophages and neutrophils, but also by chondrocytes, synoviocytes, vascular smooth cells, fibroblasts, endothelial, hepatic stellate, colonic, ductal, and airway epithelial cells. Elevated expression of YKL-40 is strongly observed in the late stages of human macrophage differentiation. The secretion of this protein is regulated inter alia by IL-6, and it requires activation of the transcription factor NF-κB, which participates in the regulation of cell proliferation and apoptosis [18,19]. On the other hand, cellular responses induced by IL-1 and TNF-α could be inhibited by YKL-40, which suggests that the induction of that glycoprotein leads to an imbalanced immune response [19]. Thus, YKL-40 can be considered one of the potential biomarkers of the chronic inflammatory progression of diseases, including endometriosis.

Prolactin (PRL) is a pituitary hormone that has a pleiotropic function, mainly during lactation and pregnancy [20]. Prolactin is also an immunomodulatory factor that has significant bioactive functions, as a hormone and a cytokine [20,21]. As a cytokine, PRL affects the proliferation and differentiation of many immune system cells [22], and influences immune system modulation, mainly by inhibiting the negative selection of autoreactive B lymphocytes [20,21]. This hormone is also an important member of the immune-neuroendocrinology network, and has been strongly associated with autoimmune diseases. Enhanced immune reactivity and a higher prevalence of autoimmune diseases are typical of women [22]. It has also been documented that serum prolactin levels are significantly elevated in women with endometriosis, however, the examinations were mainly focused on patients with endometriosis and accompanying infertility [23,24,25]. A cancer antigen (CA 125) was the last parameter to be analysed. CA 125 is a well-known peritoneal inflammation parameter and, at the same time, the most often investigated non-invasive marker of epithelial cell ovarian cancer [11,26]. CA 125 is synthesised by the coelomic epithelia, including the endometrium, fallopian tube, ovary and peritoneum. In endometriosis, CA 125 is up-regulated by stimulation of the coelomic epithelia, however, it is not an endometriosis-specific marker [26].

The results concerning the determination of the concentration of serum inflammatory parameters in women with endometriosis published by other researchers are diverse and inconclusive. Therefore, in this study, we decided to examine whether selected parameters of inflammation (IL-1β, IL-6, hs-CRP, IgG, YKL 40 and PRL) could become a set of additional diagnostic markers (all of them or some of them) supporting the diagnosis of advanced endometriosis. The obtained results were compared with the values of CA 125, which is also known as a sensitive marker of inflammation. Additionally, the study was aimed at checking whether the determination of such a set of markers might be useful for distinguishing women with endometriosis from those with benign gynecological diseases (non-endometriosis).

## 2. Results

### 2.1. The Concentrations of Inflammatory Parameters

**Figure 1:**
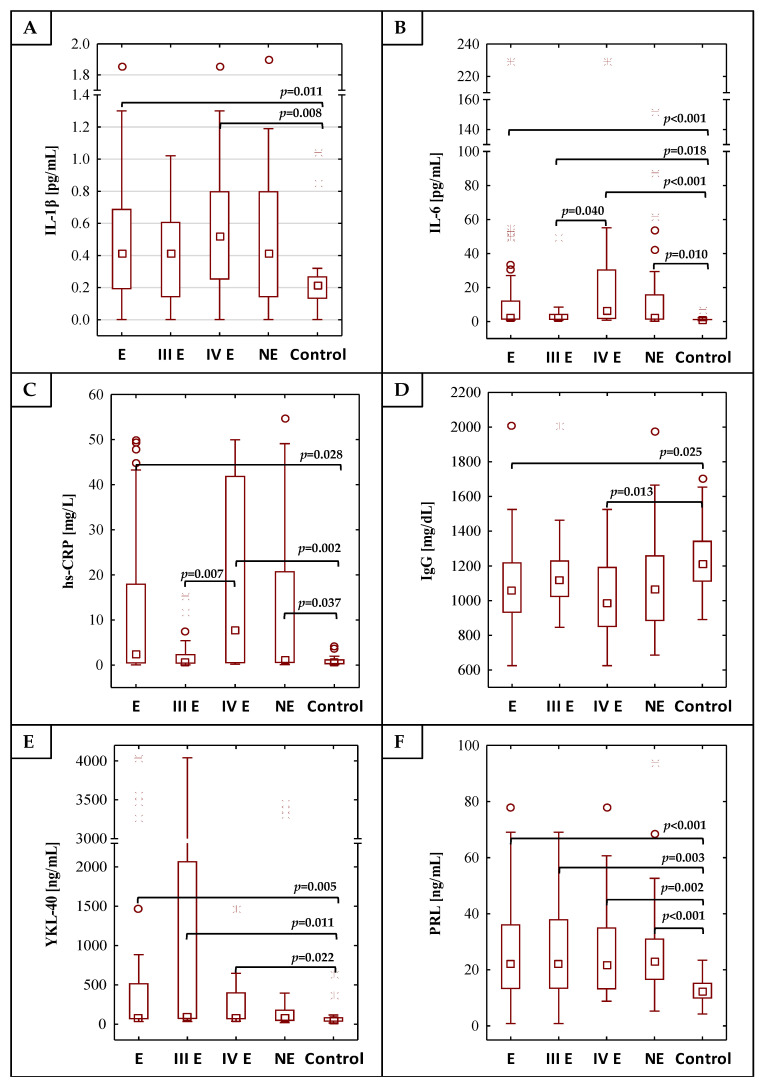

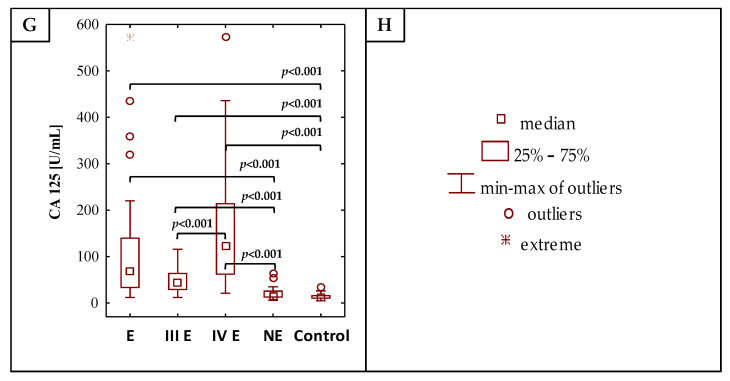
The values of determined concentrations of IL-1β, IL-6, hs-CRP, IgG, YKL-40, PRL and CA 125 (**A**–**G**), respectively), (**H**) is the legend. E—endometriosis, NE—non-endometriosis groups. Control—group of healthy volunteers, III E and IV E—third and fourth stage of endometriosis, respectively. A two-tailed *p*-Value of less than 0.05 was considered significant.

The results of the determination of IL-1β, IL-6, hs-CRP, IgG, YKL 40, PRL and CA 125 concentrations are shown in Table 1 and Figure 1. The concentrations of IL-1β, IL-6, hs-CRP, YKL-40, PRL and CA 125 were significantly higher in the E group (median values: 0.41 pg/mL, 2.42 pg/mL, 2.33 mg/L, 79.30 ng/mL, 21.88 ng/mL and 68.00 U/mL, respectively) than in the control group (median values: 0.21 pg/mL, 0.98 pg/mL, 0.52 mg/L, 49.77 ng/mL, 12.08 ng/mL and 12.20 U/mL respectively), with significance of *p* = 0.011, *p* < 0.001, *p* = 0.028, *p* = 0.005, *p* < 0.001 and *p* < 0.001, respectively. Significant differences were also observed in the concentrations of IL-6, hs-CRP and PRL when the non-endometriosis group (median values: 1.93 pg/mL, 0.99 mg/L and 22.78 ng/mL, respectively) was compared with the group of healthy women (*p* = 0.010, *p* = 0.037 and *p* < 0.001, respectively). The IgG concentrations were significantly lower in the endometriosis group (median value: 1061.21 mg/dL) in comparison to the healthy women (median value: 1210.50 mg/dL; *p* = 0.025). No significant differences were found in the concentration of serum IL-1β, IL-6, hs-CRP, IgG, YKL 40 and PRL between the endometriosis (E) and non-endometriosis (NE) groups. Only CA 125 concentrations were significantly higher in the endometriosis group in comparison to the non-endometriosis group (median values: 68.00 U/mL and 14.99 U/mL, *p* < 0.001, respectively). This parameter was the only one whose concentration was significant in the estimation of risk of advanced endometriosis, regardless of the reference group used in the model, including the non-endometriotic group—the Supplementary Tables S1 and S2.

The analysis of the values of inflammatory parameters in the moderate (III) and severe (IV) stages of the endometriosis group versus the control group showed that concentrations of IL-1β, IL-6, hs-CRP, YKL-40, PRL and CA 125 were significantly higher in the group of patients with severe endometriosis (median values: 0.52 pg/mL, 5.98 pg/mL, 7.60 mg/L, 74.62 ng/mL, 21.81 ng/mL and 123.00 U/mL, respectively) than in the control group (*p* = 0.008, *p* < 0.001, *p* = 0.002, *p* = 0.022, *p* = 0.002 and *p* < 0.001, respectively). The IgG concentration was significantly lower (*p* = 0.013) in the serum of patients with severe endometriosis (median value 982.31 mg/dL) as compared to the control group. Significantly higher concentrations of IL-6, YKL-40, PRL and CA 125 were observed in the serum of patients at a moderate stage (median values: 1.89 pg/mL, 88.61 ng/mL, 21.95 ng/mL and 45.00 U/mL) in comparison to the control group, with significances of *p* = 0.018, *p* = 0.011, *p* = 0.003 and *p* < 0.001. Between stages III and IV of endometriosis, significant differences in concentrations of IL-6 (*p* = 0.040), hs-CRP (*p* = 0.007) and CA 125 (*p* < 0.001) were observed. Stages III and IV of endometriosis also differed in CA 125 concentrations compared to the NE group (median values: 45.00 U/mL, 123.00 U/mL and 14.99 U/mL, respectively).

The significant correlations between concentration values of the determined parameters for all studied subjects are shown in Table 2.

### 2.2. ROC Curve Analysis

**Figure 2:**
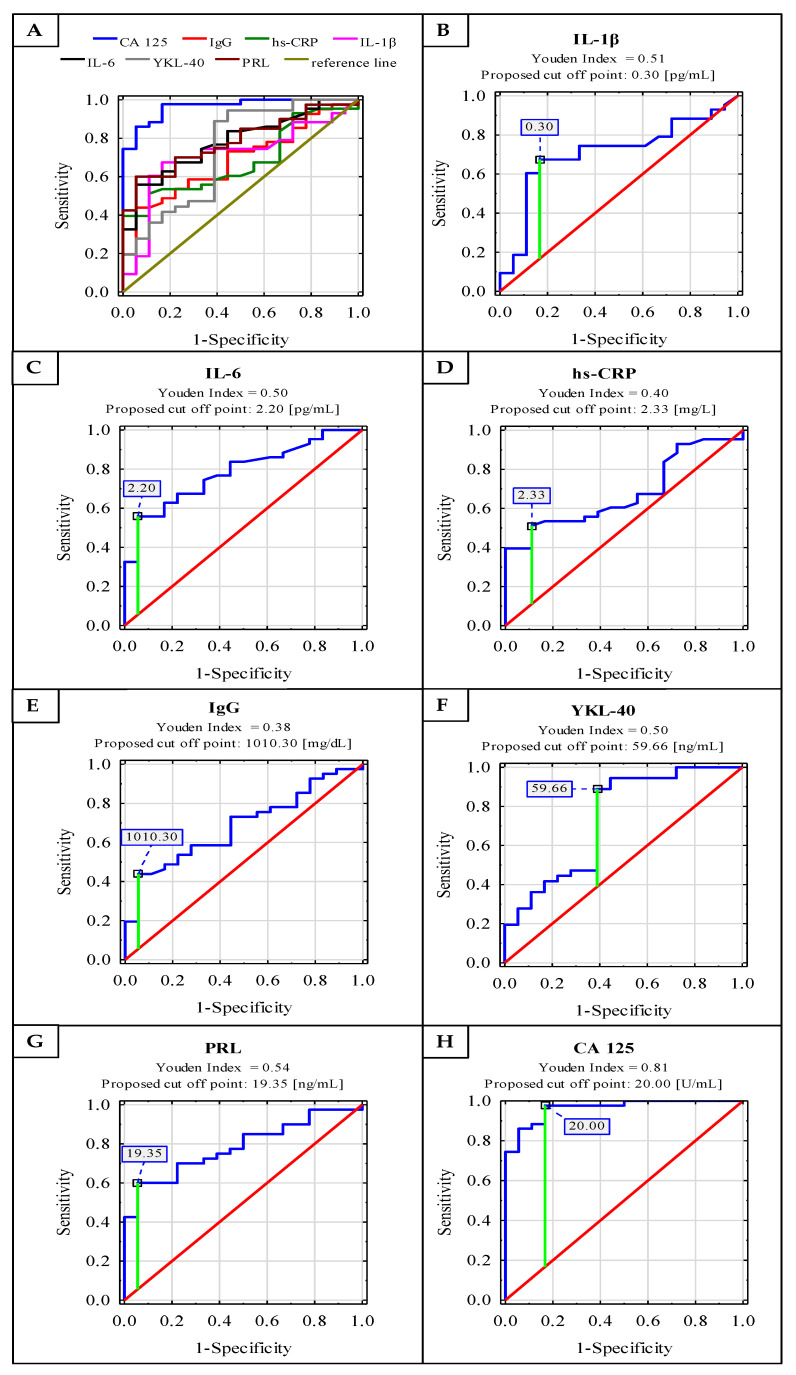

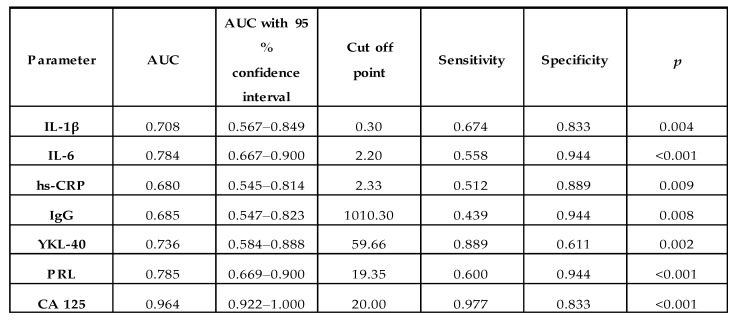
Receiver operating characteristic (ROC) curves for serum concentrations of IL-1β, IL-6, hs-CRP, IgG, YKL-40, PRL and CA 125 as markers of advanced endometriosis (**A**–**H**). Data are given as area under the ROC curve (AUC) with a 95% confidence interval.

Receiver operating characteristic (ROC) curve analysis of serum IL-1β, IL-6, hs-CRP, IgG, YKL 40, PRL and CA 125 concentrations in the endometriosis and control groups identified parameters with a sensitivity and specificity of: 0.674, 0.833 (Area under the ROC curve (AUC) 0.708—moderate clinical value); 0.558, 0.944 (AUC 0.784—moderate clinical value); 0.512, 0.889 (AUC 0.680—limited clinical value); 0.439, 0.944 (AUC 0.685—limited clinical value); 0.889, 0.611 (AUC 0.736—moderate clinical value); 0.600, 0.944 (AUC 0.785—moderate clinical value) and 0.977, 0.833 (AUC 0.964—high clinical value), respectively. For the determination of cut-off points, the Youden index method was used (Figure 2). The clinical value of a laboratory test with AUC can be defined as: 0–0.5—zero, 0.5–0.7—limited, 0.7–0.9—moderate and >0.9 high [27].

### 2.3. Cluster Analysis

**Figure 3:**
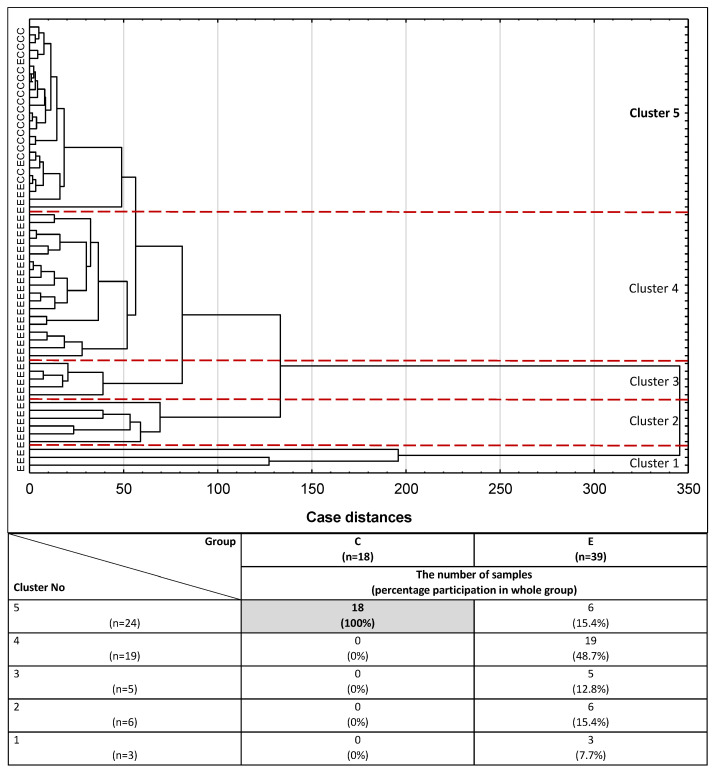
Dendrogram of cluster analysis of serum IL-6, PRL and CA 125 expression for endometriosis and control groups. E—endometriosis, C—control group. The cluster analysis was performed only for parameters with the highest AUC value and for serum samples in which the concentrations of all three parameters were determined. Each serum sample is represented by a vector of three features: IL-6, PRL and CA 125. Cluster 5 and a cut-off lane separate 100% of women from the control group and 15.4% of endometriosis patients from the rest of the endometriosis samples (Clusters 1–4).

For the purpose of conducting cluster analysis, three parameters, namely IL-6, PRL and CA 125, were selected from the panel of parameters examined, taking into account that their concentration values had to simultaneously meet the following criteria: they correlate with each other, differentiate the endometriosis group from the control group of healthy women, and have moderate or high clinical value according to the results of the ROC analysis (AUC ≥ 0.784). The potential diagnostic usefulness of the set of three inflammatory markers selected to distinguish the group of women with endometriosis from the control group of healthy subjects was verified using cluster analysis. The analysis was performed for 57 serum samples from the study participants, including all participants from the control group (*n* = 18) and 39 from the endometriosis group, for whom all selected parameters were determined. In this analysis, all subjects were divided into groups solely on the basis of similarities or differences in the concentration values of all three parameters. Although (at 100% distance) all the samples could be regarded as a single, homogenous group, on closer inspection (at 99% distance), the first cluster could be distinguished as a group of three endometriosis samples (Figure 3). Homogenous groups of endometriosis samples were also gathered in clusters 2, 3 and 4 (at 38%, 23% and 16% distance, respectively). Cluster 5 (at 14% distance) comprised 24 subjects, 18 of which were healthy women from the control group (100% of the whole group), while only six samples belonged to endometriosis patients (15.4% of the whole endometriosis group).

## 3. Discussion

The diagnosis of endometriosis remains challenging, as even the recommended standard, namely laparoscopy, has drawbacks such as invasiveness and high costs. It also has considerable limitations in the diagnosis of retroperitoneal and deep infiltrating lesions [28]. This encourages many researchers to look for inexpensive, non-invasive biomarkers for diagnosing women with suspected endometriosis.

Interleukin 1β, produced during acute phases of inflammation, is crucial to the regulation of normal immune and inflammatory responses [9]. The concentration values of serum IL-1β determined in the study (mean values: 0.50 pg/mL for endometriosis, 0.50 pg/mL for non-endometriosis and 0.27 pg/mL for the control group) are on a similar level as the results obtained by Oku et al. [29] for endometriosis and control groups (mean values: 0.12 pg/mL and 0.17 pg/mL, respectively). The results obtained in this study for serum IL-1β concentrations, as well as the findings obtained by Oku et al. [29], were several dozen times lower than those published by Jiang et al. [3], Bedaiwy et al. [30] and Malutan et al. [31]. The observed differences between our study results and the values of IL-1β concentrations reported by other authors may be due to differences in the sensitivity and/or specificity of tests used for cytokine determination as well as complex mechanisms of cytokine synthesis, and variabilities of hormonal regulation and clinical state, which differ for each woman. Moreover, Oku et al. [29], Jiang et al. [3] and Bedaiwy et al. [30] did not observe any differences in serum IL-1β concentrations between patients with endometriosis and the control group. In this study, significantly higher serum IL-1β concentrations were observed in the endometriosis group, particularly in stage IV, when compared with the control group of healthy women. This is consistent with the results of the analysis conducted by Malutan et al. [31] who observed significantly higher serum IL-1β concentrations in endometriosis than those measured in the sera of healthy women. Jiang et al. [3] and Bedaiwy et al. [30] studied the endometriosis group, in which 40% and 61% of all subjects, respectively, were at an early stage of the disease. In this study, the endometriosis group was composed of women at stages III and IV. The differences observed in IL-1β concentrations between the endometriosis and control groups and their absence in the analogous groups studied by Jiang et al. [3], Bedaiwy et al. [30] and Oku et al. [29], may also be caused by distinctions between the control groups we investigated and those examined by the authors mentioned above. It is noteworthy that the control groups studied by Oku et al. [29], Bedaiwy et al. [30] and Jiang et al. [3] consisted of women with benign gynecological diseases, and they were more similar to our NE group than to the control group of healthy women. Considering that a lack of significant differences in IL-1β concentration between groups of endometriosis and non-endometriosis (but with other gynecological diseases) was observed in this study, similarly to other studies, it may be concluded that a group of healthy women, without any diagnosed or treated diseases, should be used as a control group for the comparison of cytokine expression in endometriosis. This is due to the fact that an increase in pro-inflammatory cytokine levels accompanies not only endometriosis but many other diseases too. This conclusion seems to be confirmed by the study conducted by Malutan et al. [31], who used a group composed of healthy women without any accompanying diseases as a control group, and documented the presence of significant differences in IL-1β concentrations between the group of patients with endometriosis and the control group, as observed in this study.

Interleukin 6 is a cytokine that has pro-inflammatory properties and anti-inflammatory functions [9]. IL-6 also inhibits the growth of endometrial cells, however, these properties do not work properly in endometriotic tissue [32]. The concentrations of serum IL-6 in our endometriosis, non-endometriosis and control groups (mean values: 15.55 pg/mL, 16.23 pg/mL and 1.47 pg/mL, respectively) were similar to the concentrations published by Bedaiwy et al. [30] (median: 21.58 pg/mL for E group and 0.00 pg/mL for the control group, corresponding to our NE group), as well as to the results obtained by Kashanian et al. [33] (mean values: 30.42 pg/mL and 13.98 pg/mL, for E and the control group, comparable to our NE group, respectively). The results of serum IL-6 concentration are several times lower than those obtained by Malutan et al. [31]. The reported fluctuation of serum cytokine levels in various studies may also be due to their complicated cell-mediated mechanisms of synthesis, and differences in hormonal regulation in women [34]. Additionally, the differences observed in IL-6 concentration measurements may be caused by distinctions between the tests used and their sensitivity and/or specificity. In the study by Malutan et al. [31], about 96% of patients from the endometriosis group were at an advanced stage of disease, as in this study. The authors documented significantly higher serum IL-6 concentrations in women with endometriosis in comparison to the control group of healthy women. Nevertheless, significant differences in concentrations of the aforementioned cytokines between stages III and IV of endometriosis were not observed by the authors [31]. It should be underlined that in this study significantly higher serum IL-6 concentrations were found at stage IV of endometriosis in comparison to stage III. Together with significantly lower IL-6 concentrations in healthy subjects compared to both endometriosis groups, this may suggest that interleukin 6 concentration slowly increases from the beginning of the disease (early stages) and reaches the maximum level when endometriosis is regarded as severe. The above observation suggests that IL-6 may be a good marker of disease progression. Nevertheless, the hypothesis needs to be confirmed by future studies. Othman et al. [28] reported that serum IL-6 is a factor that differentiates women with endometriosis from healthy controls. In this study, when the determined serum IL-6 concentrations were compared between the endometriosis and non-endometriosis groups (median values: 2.42 pg/mL and 1.93 pg/mL, respectively), there were no significant differences. When analysing the discrepancies between the results from this study and other authors’ findings, it may be concluded that they could be caused by heterogeneous patient populations in the studied endometriosis groups, and subjects selected for the control group, as well as by differences in the methodologies of IL-6 concentration measurements.

The diagnostic accuracy of serum IL-6 was recently tested for endometriosis prediction. Jiang et al. [3] revealed 0.905 AUC for serum IL-6 and the proposed cut-off value was 5.99 pg/mL with 90.0% sensitivity and 93.7% specificity. The authors concluded that this parameter could be a good diagnostic tool for the detection of late-stage endometriosis and has a moderate diagnostic value for the detection of the early stages of the disease. Somigliana et al. [35] proposed a cut-off point for serum IL-6 concentration as 3.9 pg/mL with 11% sensitivity and 91% specificity. The authors concluded that IL-6 cannot be used to distinguish between patients with or without endometriosis, even in combination with CA 125. However, Somigliana et al. [35] suggested that ROC analysis of serum IL-6 levels was inadequate, and they observed low performance in detecting endometriosis even when the previously determined cut-off values were used [35]. The cut-off point for serum IL-6 in this study was 2.20 pg/mL, with 55.8% sensitivity, 94.4% specificity and 0.784 AUC. A similar threshold (2.00 pg/mL) was proposed by Bedaiwy et al. [30] with a relatively high diagnostic value, with 0.87 AUC, 90% sensitivity and 67% specificity. Malutan et al. [31] also evaluated IL-1β and IL-6 as factors for the prediction of endometriosis, which was confirmed with a specificity of 85% and 95%, respectively, while the proposed threshold values were 7 pg/mL for IL-1β and 125 pg/mL for IL-6. The authors reported that IL-1β should be analysed together with, e.g., IL-6, because measurement of only one parameter has low sensitivity (57%) [31]. In this study, with regard to ROC analysis for IL-1β, AUC was 0.708 which indicates a moderate clinical value, with a cut-off point of 0.30 pg/mL, 67.4% sensitivity and 83.3% specificity. The above results indicate that both IL-1β and IL-6 can be regarded as possible markers, providing a non-surgical way of differentiating between women with endometriosis and healthy subjects. It has already been documented that interleukin 6 is independent of the phase of the menstrual cycle, which is important from a diagnostic point of view. Othman et al. [28] did not observe the presence of correlations between stage of disease and serum IL-6 concentrations, which in their opinion makes IL-6 a qualitative marker of the presence of endometriosis rather than a factor determining the severity of the disease [28].

Serum CRP is a parameter of an acute inflammatory phase, which increases during inflammation [36]. In this study, hs-CRP concentrations had positive correlations with IL-1β and IL-6 concentrations, which may confirm that hs-CRP was produced after the stimulation of pro-inflammatory cytokines, such as IL-6 and IL-1β. Vodolazkaia et al. [14] reported that plasma CRP and hs-CRP concentrations were higher in the group with advanced (moderate-severe) endometriosis than in the control group, which is in accordance with the results obtained in this study for serum hs-CRP (mean values: 12.18 mg/L for E group and 0.96 mg/L for the control group of healthy women). Significant differences were also noted between stages III and IV of endometriosis, with higher concentrations of hs-CRP in severe endometriosis. Vodolazkaia et al. [14] demonstrated a cut-off point for plasma CRP concentration > 0.71 mg/L for stages III and IV of endometriosis, with 63.8% sensitivity, 63.7% specificity and 0.66 AUC. They observed superior diagnostic performance (higher AUC) for the high-sensitivity technique rather than for the classical method of CRP determination [14]. In this study, for hs-CRP concentrations in endometriosis, a cut-off point of 2.33 mg/L with 51.2% sensitivity and 88.9% specificity, with a moderate clinical value according to 0.680 AUC, was proposed. From the point of view of laboratory medicine, the cut-off points proposed in this and other studies are still within the physiological reference range (<10 mg/L [14]), which makes C-reactive protein one of the parameters in the panel of biomarkers useful for endometriosis diagnostics rather than a marker specific for the monitoring of disease progression. In contradiction to this, Thubert et al. [4] and Xavier et al. [37] found no differences in the medians of CRP concentration between patients with endometriosis and the control group (women without endometriosis). However, median values of hs-CRP concentrations in this study (2.33 mg/L for endometriosis and 0.52 mg/L for the group of healthy women) were similar to those obtained by Xavier et al. [37] (mean medians for all menstrual cycle phases: 1.27 mg/L for endometriosis and 0.76 mg/L for the control group). The patients belonging to the endometriosis group analysed by the authors were classified as those at advanced stages of the disease (stages III–IV ASRM) [37], which is comparable to the endometriosis group analysed in this study. Thubert et al. [4] and Xavier et al. [37] also observed no significant differences for serum CRP levels between menstrual cycle phases for either of the groups subject to analysis (the endometriosis and control groups), which indicates that CRP may be an inflammatory marker independent from the menstrual cycle, and thus useful in the diagnostics of endometriosis. In contradiction to this, Vodolazkaia et al. [14] reported discrepancies in CRP concentration between menstrual cycle phases. On the other hand, Lermann et al. [36] suggested that serum hs-CRP might be useful as a marker excluding endometriosis and Thubert et al. [4] indicated hs-CRP as an appropriate future parameter for the detection of endometriosis complications, e.g., endometrioma abscess. C-reactive protein has one more advantage, which lies in the fact that among the assessed parameters in this study it is the only parameter that is routinely used in clinical practice. As a marker of ongoing inflammation it is tested in almost all diagnostic laboratories [36]. Nowadays, CRP is measured in blood serum samples by a classical or high-sensitivity method. It seems that hs-CRP is more relevant for the diagnostics of endometriosis patients than classical measurement [14].

Immune system abnormalities, both cellular and humoral, have been demonstrated on systemic and local levels in the pathogenesis of endometriosis. The production of immunoglobulins by B lymphocytes is dependent, among other things, on the action of cytokines. Gebel et al. [38] documented that in vitro IgG2 production after the stimulation of B lymphocytes was significantly reduced among women with stages III or IV of endometriosis in comparison to the healthy controls [38]. In this study, a similar tendency in values of total serum IgG concentration in stages III and IV of the endometriosis group was observed. However, the concentrations of IgG subclasses were not determined. It is possible that the lower concentration of serum IgG in advanced stages of endometriosis may be caused by decreased expression of the IgG2 subclass. This hypothesis should be confirmed by future studies. Another possible explanation of decreased IgG concentration in severe endometriosis is that it may be associated with the follicular phase of the patients’ menstrual cycle [39], or may be caused by the treatment of patients with glucocorticoids, e.g., in allergic diseases [40]. The therapeutic strategies used can lead to the elimination of the inflammatory reaction associated with endometriosis [6]. However, we have no information about menstrual cycle phases or allergic diseases diagnosed in the groups of patients subject to the analysis. There is little information available about total immunoglobulin G concentration used as a parameter of the immune response in endometriosis. Confino et al. produced a report [41] that is consistent with the results of this study, in which patients with endometriosis have significantly lower serum IgG concentrations than healthy women do. The IgG molecule has the unique feature of initiating both pro- and anti-inflammatory reactions [16]. In this study, a significant negative correlation of IgG concentrations with the expression of hs-CRP and IL-6 was observed. Some authors have demonstrated that increased B cell production in patients with endometriosis negatively correlated with the severity of the disease. They suggested that mild endometriosis may be immunologically more active than severe disease [13,42].

Another parameter analysed in this study was YKL-40, secreted by neutrophils and macrophages in inflamed tissues, which makes it a biomarker of inflammation. However, the first available publication describing studies on YKL-40 concentration in endometriosis was published in 2010, and there is still only sparse information on this subject [43]. Tuten et al. [44] reported significantly higher serum YKL-40 concentrations in patients with endometriosis in comparison to the control group, which is in accordance with the observations of this study. Additionally, significantly higher serum YKL-40 concentrations in the endometriosis group were observed in comparison with the healthy subjects (mean values: 590.43 ng/mL and 104.12 ng/mL, respectively) in this study. In our opinion, the discrepancies in concentration values of YKL-40 obtained in various studies [44] may be caused by differences in the tests used, including their sensitivity and specificity. When stages III and IV of endometriosis were compared, lower concentrations of serum YKL-40 in the severe group were observed, however, they were insignificant. The results of the determinations presented by Tuten et al. [44] showed significantly higher serum YKL-40 concentrations in patients with advanced endometriosis when compared to the early group, and they reported that increased serum YKL-40 levels in patients with endometriosis were positively correlated with the stage of endometriosis. On the other hand, Ural et al. [45] documented that mean serum YKL-40 concentrations were 106.0 ng/mL for the endometriosis group and 52.2 ng/mL for the control group (*p* = 0.003), which was consistent with the results of YKL-40 concentrations in the endometriosis and control groups we studied. The aforementioned results may suggest that YKL-40 could be a useful parameter for the identification of women with endometriosis, but in the form of a reliable exclusion marker rather than as a diagnostic marker of early disease detection or monitoring its progression. Nevertheless, this theory needs to be confirmed by further investigations.

Johansen et al. [46] reported that concentrations of YKL-40 in the serum of healthy subjects were maintained at around 40 ng/mL, and were not connected with the circadian rhythm. In this study, the cut-off point was proposed at 59.66 ng/mL for diagnosed endometriosis, with 88.9% sensitivity, 61.1% specificity and 0.736 AUC [46]. Positive correlations between YKL-40 and pro-inflammatory cytokine (IL-1β), which may confirm the inflammatory connection of the glycoprotein, were shown in this study. Taking into account the above-mentioned information and the results of this study, it seems that YKL-40, together with IL-1β, can be used as additional markers of inflammation, helpful in the diagnosis of endometriosis.

The functions of prolactin include, among others, immunological reactivity [47] ], and PRL as an immunomodulating factor modulates the immune system [20,21]. In this study, significantly higher concentrations of serum prolactin were observed in the endometriosis (*p* < 0.001) and non-endometriosis groups (*p* < 0.001), in comparison to the healthy women from the control group (median values: 21.88 ng/mL, 22.78 ng/mL and 12.08 ng/mL, respectively). These differences were also visible for the III (*p* = 0.003) and IV (*p* = 0.002) stages of endometriosis analysed separately, when compared to the control group. The results obtained in this study for serum PRL are in accordance with those published by Lima et al. [47], who reported significantly higher serum prolactin levels in infertile women at stages III-IV of endometriosis than in healthy controls. The authors suggested that higher levels of serum prolactin may be associated with the progression of endometriosis [47]. Esmaeilzadeh et al. [24] showed significant correlations between serum PRL concentrations and stages of endometriosis as compared to the healthy controls (*p* = 0.01) [24]. The significant positive correlations between PRL concentrations and the concentrations of inflammatory parameters, IL-1β, IL-6, hs-CRP and CA 125 observed in this study may be explained by the fact that PRL secretion can be regulated inter alia by cytokines, such as IL-1 and IL-6 [22].

In the meta-analysis performed by Gao et al. [23] concerning serum prolactin concentrations, the sensitivity and specificity in endometriosis were 45% and 92%, respectively. The authors also observed that the serum prolactin cut-off point of 14.8 ng/mL had higher sensitivity (44%), than a cut-off threshold of 20.0 ng/mL (21%; *p* < 0.05). It was highlighted that despite the high specificity, the sensitivity of serum prolactin for the diagnosis of endometriosis remained unacceptably low [23]. The prolactin cut-off point proposed for endometriosis diagnostics in this study is 19.35 ng/mL, with a sensitivity of 60% and specificity of 94.4%. AUC had a moderate clinical value of 0.785.

CA 125 is a glycoprotein that has been widely examined and considered to be a useful diagnostic tool in endometriosis [48,49,50,51,52]. In this study, significantly higher concentrations of serum CA 125 in the endometriosis group were observed in comparison to the healthy control (median values: 68.00 U/mL and 12.20 U/mL, respectively; *p* < 0.001), even for the non-endometriosis group (median value: 14.99 U/mL; *p* < 0.001). Ding et al. [53] also observed significantly higher levels of serum CA 125 in women with advanced endometriosis than in women with non-endometriosis benign ovarian cysts (*p* < 0.0001). Similar results were shown by Kashanian et al. [33], who reported that the concentrations of serum CA 125 in moderate to severe endometriosis were significantly higher than in the control group (*p* = 0.022). Moreover, significantly higher serum CA 125 levels in stage IV rather than in stage III of endometriosis (*p* < 0.001) were demonstrated in this study. Maiorana et al. [54] documented an association between endometriosis identification based on rAFS classification and serum CA 125 level, which was not confirmed by Kim et al. [48] who performed a similar analysis on a group of 419 women with advanced endometriosis. In this study, serum CA 125 concentrations were on a similar level at stages III and IV of endometriosis. Moreover, there were no differences in CA 125 concentration between the NE group and healthy women.

The sensitivity and specificity of serum CA 125 in the diagnostics of endometriosis vary in the studies carried out by various authors [reviewed in [55]]. In this study, the cut-off point for serum CA 125 for the diagnosis of advanced stages of endometriosis was proposed at 20.00 U/mL with high clinical value: 0.964 AUC, 97.7% sensitivity and 83.3% specificity. A similarly high clinical value of CA 125 for determining endometriosis was documented by Ding et al. [53], who proposed 0.924 AUC, 82.3% sensitivity and 90.0% specificity, and cut-off value at 30.75 IU/mL. On the other hand, Kashanian et al. [33] suggested a cut-off point of 35 IU/mL with 44.76% sensitivity, 94.73% specificity and 0.69 AUC for CA 125. Maiorana et al. [54] in their study also demonstrated that serum CA 125 concentration is a good parameter for the detection of advanced endometriosis. They reported 76% sensitivity and 94% specificity for stages III–IV. Nisenblat et al. [55] in a meta-analysis assessed CA 125 at different thresholds, but the cut-off interval of >16.0–17.6 U/mL reached their criteria of a rule-in test at the highest possible level, with a mean sensitivity of 56% and mean specificity of 91%. In the second meta-analysis performed by Hirsch et al. [26], the authors concluded that concentration of CA 125 ≥ 30 U/mL in a group of symptomatic women is highly specific to the diagnostics of endometriosis and it could act as a rule-in test, showing a sensitivity of 52.4% and specificity of 92.7%, but concentrations below that point do not exclude disease [26]. These differences in the two meta-analyses may be due to different inclusion criteria, definitions of a rule-in test and cut-off intervals.

In routine medicine, CA 125 is often used as a marker of gynecological diseases, including advanced endometriosis. In this study, significant correlations between CA 125 concentrations and concentrations of the investigated inflammatory markers (IL-1β, IL-6, hs-CRP and prolactin) were observed. Out of all the examined parameters, only three were selected for cluster analysis, simultaneously fulfilling the following criteria: they should be correlated with each other, differentiate the endometriosis group from the control group of healthy women, and have moderate or high clinical values (AUC ≥ 0.784). Such analysis determines whether the set of concentration values of CA 125, IL-6 and PRL can be used to differentiate serum samples in a way that reflects clinical characteristics typical for endometriosis. Out of the five clusters formed, only cluster no. 5 differed with regard to the clinical characteristics of the women, gathering together all the samples of healthy women (*n* = 18) and six samples from the endometriosis group (15.4%). This finding supports the hypothesis that the proposed set of inflammatory parameters, with particular emphasis on those that were selected for cluster analysis, not only may provide valuable information for better identification of the advanced stage of endometriosis, but also should be taken into account as additional markers useful for the diagnostics of endometriosis.

**Limitations of the Study**

1. Lack of a representative early-stage endometriosis group, which makes it impossible to verify the parameters analysed in this study and evaluate them as useful biomarkers for the early stages of endometriosis.
2. The broad range of concentration values obtained for all the parameters subject to analysis has an influence on the results of statistical analysis and makes it difficult to draw unequivocal conclusions.
3. Lack of the peritoneal fluid needed to compare the concentrations of inflammatory parameters with their expression in the serum.

**Strengths of the Study**

1. The proposed panel of easily measurable serum markers of inflammation (especially IL-6, PRL and CA 125) may be helpful in the differentiation of the advanced stage of endometriosis, and for its diagnostics.
2. The set of parameters analysed in this study could be a useful clinical tool to identify women with a high risk of severe endometriosis development, who could qualify for a laparoscopy procedure.
3. The results of this study encourage further research concerning a noninvasive, easily measurable diagnostic panel of inflammatory markers, with potential clinical utility in women with advanced endometriosis.
4. The study showed that the non-endometriosis group, suffering from benign gynecological diseases, did not seem to be a proper comparative population representing disorders with similar inflammatory parameters compared to women with advanced endometriosis.

## 4. Materials and Methods

### 4.1. Patients

The serum samples of women with endometriosis (E, *n* = 43) and without endometriosis (comparative group; non-endometriosis, NE, *n* = 35) were collected at the Department of Oncological Gynecology, Wroclaw Comprehensive Cancer Center (Wroclaw, Poland). The control group was composed of healthy female volunteers (control group, *n* = 18) in their reproductive years. The two first groups of patients (E and NE) had undergone surgical interventions, mainly laparoscopy, and, following histological verification, they were assigned to the proper group. Women with endometriosis were also classified based on the extent and severity of the disease according to rAFS classification. The women in the NE group were histologically confirmed with leiomyomas, benign ovarian cysts or with severe dysplasia—CIN 3 (cervical intraepithelial neoplasia grade 3). The control group consisted of healthy, non-pregnant women, without any gynecological problems or indicators of illness, and with no history of symptoms related to endometriosis.

The blood samples were collected from participants in the morning in fasting condition, with the use of a clot activator. After 30 min at room temperature, the tubes were centrifuged at 2500 rpm for 10 min in order to obtain serum. The serum samples were stored at −80 °C before analysis. This study was approved by the Bioethics Committee of Wroclaw Medical University (No. 231/2019 and No. 685/2019). All subjects gave written and informed consent prior to their participation in the research. All the examinations were carried out in accordance with the manufacturer’s instructions.

Patient demographics: Among 43 women with histologically confirmed endometriosis, based on rAFS classification, the following two groups can be distinguished: 20 women with moderate and 23 with severe endometriosis (stages III and IV, respectively). All participants were of a similar age and had comparable body mass indexes (BMI)—Table 3.

### 4.2. Assays

Interleukin 1β, interleukin 6 and YKL-40 were determined with commercially available ELISA tests, according to the recommendations of the manufacturer. Human IL-1β ELISAPRO kit (MABTECH AB, Nacka Strand, Stockholm, Sweden) was used for Interleukin 1β concentrations, High Sensitivity ELISA kit (The Covalab, Villeurbanne, France) for interleukin 6 and Human Chitinase-3-like Protein 1 ELISA Kit (Bioassay Technology Laboratory, Shanghai, China) for YKL-40 concentrations. In order to determine concentrations of the aforementioned inflammatory parameters, Mindray-96A reader was used. C-reactive protein and IgG concentrations were measured using the immunoturbidimetric method (highly sensitive for CRP, U-hs, DiaSys Diagnostic Systems GmbH, Germany and immunoglobulin G FS, DiaSys Diagnostic Systems GmbH, Holzheim, Germany, respectively), using the biochemical analyser Konelab 20i^®^ (ThermoScientific, Vantaa, Finland). CA 125 and prolactin concentrations were measured by Cobas^®^ 6000 analyser (Roche, Mannheim, Germany).

### 4.3. Statistical Analysis

The normality of distribution of all parameters was analysed by the Shapiro–Wilk test and all values of the examined parameters were presented as mean ± SD (SD—standard deviation) and as median with interquartile range (Q1–Q3) on the graphs. The U Mann–Whitney test was applied for the purpose of comparison of all parameters between the control group versus endometrial and non-endometrial groups. Spearman rank correlation was used to check correlations between the measured parameters. The diagnostic significance of the tested inflammatory parameters was analysed using receiver operating characteristic (ROC) curves. Additionally, cluster analysis was performed for serum samples, in which all parameters were determined. In that analysis, the results were presented as a dendrogram, starting from one cluster in which all subjects (patients and controls) were gathered. Next, the subjects were clustered. Patients presenting similarities in terms of the values of all the analysed traits were grouped together, and those with different values formed a separate cluster. In general, it was assumed that the greater the distance of separation, the greater differences in subject characteristics. The similarities between samples were calculated using a Euclidean metric on the original data points, with no reference to the clinical status of the samples. For statistical analysis, undetectable amounts of measured target protein were considered to be 0.001. Univariate and multivariate logistic regression was performed, in which endometriosis was always the modeled group. The results of univariate (Supplementary Table S1) and multivariate (Supplementary Table S2) logistic regression models are summarized in Supplementary Materials. Statistical analysis was performed using Statistica PL version 13.3 (StatSoft Polska Sp. z o.o., Warsaw, Poland). The *p*-Value < 0.05 was considered significant.

## 5. Conclusions

The diagnostics of endometriosis present a challenging task, especially when non-invasive methods are considered. The development of the disease is associated with complex inflammatory processes, the immunological determinants of which may be detected in peripheral blood serum. Such disorders are clearly expressed in the advanced stages of endometriosis. Thus, a well-defined panel of biomarkers could be helpful during endometriosis diagnostics, supplementing thorough medical interviews and tests, including imaging. The panel of serum biomarkers proposed and examined in this study includes inflammatory markers IL-1β, IL-6, hs-CRP, YKL-40 and PRL, which were analysed in comparison to the well-known inflammatory marker CA 125. In our opinion, the proposed panel of parameters, particularly IL-6, PRL and CA 125, could be a useful clinical tool to identify women with a high risk of endometriosis development, due to the enhanced expression of inflammatory biomarkers when compared to the healthy population. Future studies are required to evaluate the clinical utility and effectiveness of this set of markers for standard endometriosis diagnostics.

## Figures and Tables

**Table 1:** The concentrations of selected parameters in the studied groups.

	E N = 43	III E N = 20	IV E N = 23	NE N = 35	CONTROL N = 18
	MEAN ± SD	MEAN ± SD	MEAN ± SD	MEAN ± SD	MEAN ± SD
**IL-1β(pg/mL)**	0.50 ± 0.39 *p* = 0.011 *	0.43 ± 0.30	0.57 ± 0.44 *p* = 0.008 *	0.50 ± 0.41	0.27 ± 0.26
**IL-6 (pg/mL)**	15.55 ± 36.93 *p* < 0.001 *	5.07 ± 10.69 *p* = 0.018 *	24.67 ± 48.16 *p* < 0.001 * p = 0.040 **	16.23 ± 31.53 *p* = 0.010 *	1.47 ± 1.48
**hs-CRP (mg/L)**	12.18 ± 17.73 *p* = 0.028 *	2.57 ± 4.24	20.53 ± 20.68 *p* = 0.002 * p = 0.007 **	11.80 ± 18.59 *p* = 0.037 *	0.96 ± 1.19
**IgG (mg/dL)**	1086.04 ± 267.85 *p* = 0.025 *	1161.54 ± 250.87	1014.14 ± 269.39 *p* = 0.013 *	1111.94 ± 293.32	1237.28 ± 215.91
**YKL-40 (ng/mL)**	590.43 ± 1118.07 *p* = 0.005 *	1007.16 ± 1557.98 *p* = 0.011 *	257.05 ± 351.05 *p* = 0.022 *	449.73 ± 1019.59	104.12 ± 154.07
**PRL (ng/mL)**	26.80 ± 17.33 *p* < 0.001 *	27.58 ± 17.34 *p* = 0.003 *	26.10 ± 17.72 *p* = 0.002 *	27.55 ± 18.30 *p* < 0.001 *	12.84 ± 4.86
**CA 125 (U/mL)**	109.56 ± 118.22 *p* < 0.001 * p < 0.001 ***	47.55 ± 26.51 *p* < 0.001 * p < 0.001 ***	163.48 ± 139.78 *p* < 0.001 * p < 0.001 ** p < 0.001 ***	21.84 ± 16.20	14.28 ± 7.40

Significant differences: * versus control group, ** versus stage III of endometriosis, *** versus NE group. E—endometriosis group, III E—moderate group of endometriosis (stage III according to rAFS classification), IV E—severe group of endometriosis (stage IV according to rAFS classification), NE—non-endometriosis group. A two-tailed *p*-Value of less than 0.05 was considered significant.

**Table 2:** The correlations between concentrations of determined parameters.

Parameter	IL-1β [pg/mL]	IL-6 [pg/mL]	hs-CRP [mg/L]	IgG [mg/dL]	YKL-40 [ng/mL]	PRL [ng/mL]
IL-1β (pg/mL)						
IL-6 (pg/mL)	R = 0.383 *p* < 0.001					
hs-CRP (mg/L)	R = 0.337 *p* < 0.001	R = 0.610 *p* < 0.001				
IgG (mg/dL)	NS	R = −0.475 *p* < 0.001	R = −0.448 *p* < 0.001			
YKL-40 (ng/mL)	R = 0.243 *p* = 0.008	NS	NS	R = 0.186 *p* = 0.043		
PRL (ng/mL)	R = 0.371 *p* < 0.001	R = 0.230 *p* = 0.008	R = 0.286 *p* < 0.001	NS	NS	
CA 125 (U/mL)	R = 0.181 *p* = 0.043	R = 0.265 *p* = 0.003	R = 0.316 *p* < 0.001	R = −0.235 *p* = 0.009	NS	R = 0.325 *p* < 0.001

A two-tailed *p*-Value of less than 0.05 was considered significant; NS—not significant.

**Table 3:** The characteristics of study groups.

	E	III E	IV E	NE	CONTROL
	MEAN ± SD	MEAN ± SD	MEAN ± SD	MEAN ± SD	MEAN ± SD
N	43	20	23	35	18
Age [years]	35 ± 8	33 ± 7	38 ± 8	38 ± 8	40 ± 8
BMI [kg/m^2^]	24.73 ± 3.43	23.80 ± 2.78	25.54 ± 3.79	25.91 ± 3.00	24.51 ± 3.99

BMI—body mass index, E—endometriosis group, III E—moderate group of endometriosis (stage III according to rAFS classification), IV E—severe group of endometriosis (stage IV according to rAFS classification), N—participants number, NE—non-endometriosis group.

## Data Availability

The data presented in this study are available on reasonable request from the corresponding author.

## Supplementary Materials

The following are available online at https://www.mdpi.com/1422-0067/22/5/2295/s1.

## Abbreviations

AUC	Area under the ROC curve
BMI	Body mass index
C	Control group
CRP	C-reactive protein
E	Endometriosis group
hs-CRP	high sensitive CRP
IgG	Immunoglobulin G
IL-1β	Interleukin 1β
IL-6	Interleukin 6
NE	Non-endometriosis group
PRL	Prolactin
rAFS	revised American Fertility Society classification
ROC	Receiver operating characteristic
SD	Standard deviation
YKL-40	Chitinase-3-like protein 1
III E	Moderate group of endometriosis (stage III according to rAFS classification)
IV E	Severe group of endometriosis (stage IV according to rAFS classification)
