# Supplementary material for: Diagnostic Significance of Selected Serum Inflammatory Markers in Women with Advanced Endometriosis
Source: Int J Mol Sci. 2021 Feb 25;22(5):2295. doi: 10.3390/ijms22052295 (PMC7956504; doi:10.3390/ijms22052295)
Supplement: Supplementary file 1 [file ijms-22-02295-s001.pdf]

Table S1. The results of univariate logistic regression models.

Parameter / Analyzed effect	CA 125		PRL		IgG		hs-CRP		IL-1β		IL-6		YKL-40	
	**OR (95%)**	***p*-value**	**OR (95%)**	***p*-value**	**OR (95%)**	***p*-value**	**OR (95%)**	***p*-value**	**OR (95%)**	***p*-value**	**OR (95%)**	***p*-value**	**OR (95%)**	***p*-value**
**E vs.** C	**1.219 (1.074–1.383)**	**0.002**	**1.141 (1.037–1.255)**	**0.007**	0.998 (0.995–1.00)	0.051	1.284 (0.927–1.778)	0.133	**12.079 (1.212–120.408)**	**0.034**	1.462 (0.953–2.244)	0.082	1.002 (0.999–1.006)	0.187
**III E vs.** C	**1.198 (1.060–1.354)**	**0.004**	**1.136 (1.026–1.258)**	**0.014**	0.999 (0.996–1.001)	0.326	1.252 (0.898–1,746)	0.186	7.837 (0.617–99.551)	0.112	1.415 (0.926–2.164)	0.109	1.002 (0.999–1.004)	0.252
**IV E vs.** C	**1.239 (1.008–1.524)**	**0.042**	**1.174 (1.035–1.332)**	**0.013**	**0.996 (0.993–0.99)**	**0.016**	1.393 (0.912–2.129)	0.125	**15.435 (1.269–187.794)**	**0.032**	1.443 (0.938–2.220)	0.096	1.003 (0.999–1.007)	0.130
**E vs.** NE	**1.071 (1.029–1.114)**	**0.001**	0.998 (0.972–1.024)	0.855	1.000 (0.998–1.001)	0.686	1.001 (0.976–1.026)	0.928	0.987 (0.319–3.050)	0.982	0.999 (0.986–1.013)	0.930	1.000 (1.000–1.001)	0.597
**III E vs.** NE	**1.063 (1.020–1.108)**	**0.004**	1.000 (0.969–1.032)	0.995	1.001 (0.999–1.003)	0.524	0.938 (0.872–1.008)	0.081	0.560 (0.119–2.641)	0.464	0.970 (0.928–1.013)	0.173	1.00 (1.000–1.001)	0.162
**IV E vs.** NE	**1.076 (1.027–1.127)**	**0.002**	0.995 (0.965–1.027)	0.769	0.999 (0.997–1.001)	0.220	1.023 (0.996–1.051)	0.102	1.426 (0.412–4.937)	0.576	1.006 (0.992–1.020)	0.430	1.000 (0.999–1.001)	0.429
**E vs.** C+NE	**1.089 (1.046–1.133)**	**<0.001**	1.015 (0.990–1.041)	0.228	0.999 (0.997–1.001)	0.225	1.015 (0990–1.040)	0.241	1.687 (0.584–4.870)	0.334	1.004 (0.991–1.018)	0.510	1.000 (1.000–1.001)	0.212

Statistically significant data (*p*-value of less than 0.05) are shown in red bold. C – control group, E – endometriosis group, III E – moderate group of endometriosis (stage III according to rAFS classification), IV E – severe group of endometriosis (stage IV according to rAFS classification), NE – non-endometriosis group OR – Odds Ratio.

Table S2. Backward stepwise regression after verification of the collinearity of the predictors (parameters).

Parameters	Odds Ratio (OR)	95% Confidence Interval (CI)	*p*-value
**E vs.** C			
**CA 125**	**1.280**	**1.085 – 1.511**	**0.004**
**III E vs.** C			
**CA 125**	**1.388**	**1.030 – 1.869**	**0.031**
**hs-CRP**	1.978	0.803 – 4.872	0.138
**IV E vs.** C			
**CA 125**	**1.239**	**1.008 – 1.524**	**0.042**
**E vs.** NE			
**CA 125**	**1.075**	**1.031 – 1.122**	**0.001**
**III E vs.** NE			
**CA 125**	**1.080**	**1.024 – 1.140**	**0.005**
**IL-6**	0.969	0.924 – 1.015	0.183
**IV E vs.** NE			
**CA 125**	**1.102**	**1.033 – 1.176**	**0.003**
**PRL**	**0.891**	**0.796 – 0.998**	**0.047**
**E vs.** C+NE			
**CA 125**	**1.094**	**1.048 – 1.142**	**<0.001**

Statistically significant data (*p*-value of less than 0.05) are shown in red bold. C – control group, E – endometriosis group, III E – moderate group of endometriosis (stage III according to rAFS classification), IV E – severe group of endometriosis (stage IV according to rAFS classification), NE – non-endometriosis group OR – Odds Ratio.
